# Power reflectance testing in newborns and infants^☆^^☆☆^

**DOI:** 10.1016/j.bjorl.2015.08.008

**Published:** 2015-09-07

**Authors:** Ticianna Garambone de Cerqueira Lima, Helena Maria Gonçalves Becker, Celso Gonçalves Becker, Daniele Barreto da Cunha Ferreira, Camilo Brandão de Resende, Roberto Eustáquio Santos Guimarães

**Affiliations:** a Universidade Federal de Minas Gerais (UFMG), Belo Horizonte, MG, Brazil; b Medicine School, Universidade Federal de Minas Gerais (UFMG), Belo Horizonte, MG, Brazil; c Newborn Hearing Screening Team, Hospital das Clínicas, Universidade Federal de Minas Gerais (UFMG), Belo Horizonte, MG, Brazil; d Graduate Program in Aeronautical Engineering and Mechanics, Instituto Tecnológico de Aeronáutica (ITA), São José dos Campos, SP, Brazil; e Universidade de São Paulo (USP), Ribeirão Preto, SP, Brazil

**Keywords:** Hearing tests, Middle ear, Neonatal screening, Testes auditivos, Orelha média, Triagem neonatal

## Abstract

**Introduction:**

Auditory screening in newborns allows for detection of hearing problems early in life. However, middle ear diseases can make the diagnosis more difficult.

**Objective:**

To evaluate the power reflectance test as an indicator of the middle ear disease and to compare it to tympanometry.

**Methods:**

Case study evaluating 105 newborns and infants who participated in the audiology screening in 2013. The following exams were performed: transient otoacoustic emissions, power reflectance, and tympanometry.

**Results:**

In the optoacoustic emission evaluation, approximately 95% of the subjects passed the test. The specificity of power reflectance in all frequencies studied ranged from 75.3% to 95.9%, and that of tympanometry at 1000 Hz ranged from 83% to 87.2%; there was agreement among these exams.

**Conclusion:**

The outcome of power reflectance tests at 2000 Hz and 3000 Hz showed a correlation with tympanometry and otoacoustic emissions, and these were the most appropriate frequencies to determine middle ear disease through power reflectance measurement. It was also observed that values of power reflectance above reference levels suggested the presence of fluid in the middle ear, and thus a conductive hearing loss.

## Resumo

**Introdução:**

A triagem auditiva neonatal nos permite identificar precocemente alterações auditivas. Entretanto, doenças da orelha média podem dificultar o diagnóstico.

**Objetivo:**

Avaliar o exame de reflectância de potência como um indicador de doença de orelha média e compará-lo com a timpanometria.

**Método:**

Estudo de casos em que foram avaliados 105 neonatos e lactentes que fizeram parte da triagem auditiva em 2013. Foram realizados os seguintes exames: emissões otoacústicas transientes, reflectância de potência e timpanometria.

**Resultados:**

Na avaliação das emissões otoacústicas, cerca de 95% passaram. A especificidade da reflectância de potência em todas as frequências pesquisadas variou de 75,3% até 95,9% e da timpanometria em 1.000 Hz variou de 83% até 87,2% e houve concordância entre esses exames.

**Conclusão:**

O resultado do exame de reflectância nas frequências de 2.000 Hz e 3.000 Hz apresentou correlação com os resultados da timpanometria e com as emissões otoacústicas, e foram estas as frequências mais adequadas para a determinação de doença de orelha média pela reflectância de potência. Observou-se que valores da reflectância de potência acima dos padrões sugerem presença de líquido em orelha média, portanto uma alteração condutiva.

## Introduction

Deafness affects one to three per 1000 children at birth, according to the Joint Committee on Infant Hearing.^1^ Of all birth defects, deafness is the most common. Early diagnosis of hearing loss and its type allows appropriate care of children with disabilities. Transient otoacoustic emissions (TOAE) are used in obtaining diagnosis. When performing otoacoustic emissions, cochlear function is assessed.^2^ For these emissions to reach the cochlea, they must pass through the external and middle ear. If there is any disease affecting their course, the emissions will not produce a response, and the cochlea will not be evaluated properly.^3^ Therefore, other tests are used to assist in the assessment of the origin of hearing impairment. Tympanometry is the gold standard for evaluation of the middle ear; this test reveals tympanic membrane-auditory ossicle mobility.^4^ Tympanometry with the use of high frequency (1000 Hz) is recommended for diagnostic testing of children under 4 months, according to Joint Committee on Infant Hearing,^1^ because this technique is more sensitive to middle ear dysfunctions compared to 226 Hz tympanometry.^5^, ^6^, ^7^

Wideband reflectance (WBR) is a new technique for assessing middle ear that has been studied in the last decade. WBR represents the acoustic energy incident on tympanic membrane (TM); it is reflected and returns to the external auditory canal, thus allowing an evaluation of the middle ear as a whole.^8^ WBR includes a set of measures that can be used to represent the acoustic behavior of the ear, *e.g.*, power reflectance (PR), absorption, impedance, transmittance, admittance, sound pressure level, and sound intensity. In this study, PR was evaluated at frequencies of 1000; 2000; 3000, and 4000 Hz, since the power reflectance measured at any point in the auditory meatus is equal to PR in the tympanic membrane.^9^, ^10^

The overall aim of this study was to analyze the correlation of PR with tympanometry in the detection of middle ear change in newborns presenting TOAE.

## Methods

This was an observational cross-sectional study that evaluated TOAE, PR, and tympanometry tests.

The sample consisted of 105 infants under the age of 40 days, of both genders (58 female and 47 male), all attendees of the Neonatal Hearing Screening Program (NHSP) from July to September 2013. All at-term newborns with no risk factor for hearing loss according to the Joint Committee on Infant Hearing,^1^ referred by NHSP for the first test, and whose parents or guardians consented to the inclusion of data from their exams in the study and signed an informed consent were included in the study. Newborns whose anamnesis protocol and results did not contain all information necessary to conduct the research were excluded. The sample size calculation was conducted through population average, which determines the minimum size of a sample to estimate a statistical parameter,^11^ based on a sampling error of 5%, with a significance level of 5% and standard deviation, and considering a mean of 7% of abnormal tests.

This study was approved by the Research Ethics Committee, CAAE No. 0673.0.203.000-11.

Hearing screening was conducted in the Audiology Department and consisted of anamnesis, behavioral tests, and TOAE. Neonates were referred to the Otolaryngology Department for performance of PR and tympanometry by the investigator. The babies were in natural sleep during the tests. The first ear to be evaluated was chosen depending on the position of the child in the mother's lap.

TOAE were captured at frequencies of 2000 Hz, 3000 Hz, and 4000 Hz using an ILO292 USB Echoport device (Otodynamics™). The emission registration protocol adopted used non-linear click stimuli at an intensity of 80 dB SPL with a testing window of 12 ms, with 512 stimuli. TOAE were considered present when reproducibility ≥70% and S/N (signal/noise) ratio ≥6 dB.

PR was performed using a middle ear acoustic energy analyzer (Middle Ear Power Analyzer [MEPA], version 3.3 – Mimosa Acoustics™) with hardware incorporated into the DPOAE Measurement System (Starkey™) at frequencies of 1000 Hz, 2000 Hz, 3000 Hz, and 4000 Hz, with chirp stimulation^12^ and a rubber-type probe (Mimosa Study standard).^13^

Chirp stimulus is the best for most patients, as it has better resolution of frequency, is faster, and works best in a quiet environment. The stimulus pressure level was 0–80 dB SPL and the measurement time was 10 s.^12^

Power reflectance calculation depends on the ear canal area. Huang et al.^14^ showed that WBR measurements require that Thevenin^15^ equivalents of the acoustic measurement system are determined with loads measuring between 10% and 15% of the actual diameter of the ear canal. The Mimosa Acoustics System^13^ calculates the ear canal area based on the probe tip diameter; this system uses the Thevenin calibration parameter.^15^ With this system, the area of the ear canal is estimated as being 4.5 mm (rubber tip cavity) or 7.5 mm (foam tip cavity); this study used the rubber tip. The diameter of the ear canal of neonates was measured with 4.4 mm diameters^16^, ^17^; thus, calibrations using the “rubber tip cavity” Mimosa method (used in this study) were appropriate.

The following patterns were used to determine which ears passed the PR test:

At 1000 Hz: 0.2–0.75, or 20–75%
At 2000 Hz: 0.1–0.50, or 10–50%
At 3000 Hz: 0–0.50, or 0–50%
At 4000 Hz: 0–0.70, or 0–70%

Tympanometry at 1000 Hz was obtained using an Impedance Audiometer AT 235 (Interacoustics™) device in the pressure recording protocol; it allowed a variation of +300 PA to −300 PA, and the type A pattern was adopted as normal, with alterations in the curves C, B, and PD.^18^, ^19^

This study used Excel™ for preparation of a database with information from right and left ears of the 105 patients included in the project. A “Passed” (*P*) test occurred when the baby presented otoacoustic emissions in all evaluated frequencies. PR was evaluated at frequencies of 1000, 2000, 3000, and 4000 Hz, and the result of each frequency was analyzed separately. Analyses were performed with R software version 2.7.1, and with Epi Info software version 6.04, both of which are public domain. A 5% significance level was set.

Considering that in this study more than two tests were evaluated, it was necessary to use multiple *κ* coefficient for estimating the degree of correlation between tests.^20^ The classifications of calculated *κ* coefficients^21^ were as follows: low (<0.20); reasonable (0.21–0.40); moderate (0.41–0.60); good (0.61–0.80), and very good (0.80–1.00). For tympanometry and PR tests, specificity was calculated considering the TOAE test as the gold standard. Specificity was considered as the rate of patients who passed the test of interest and who also passed TOAE.

## Results

In the overall assessment of TOAE, that is, those infants who passed the three frequencies evaluated, it was found that on the right side, 97 (92.4%) passed the test and eight (7.6%) failed; and on the left side, 96 (91.4%) passed the test and nine (8.6%) failed.

It was found that, in the tympanometry for the right side, 86 (85.1%) infants had a normal curve, 15 (14.9%) exhibited alterations in the curve, and there was no information for four children. On the left side, 81 (79.4%) children had a normal curve, 21 (20.6%) exhibited alterations in the curve, and there was no information for three children. Cases without information were those with no curve formation, or for which there was no bilateral seal.

Table 1 shows the description of PR test for right and left sides of the 105 infants evaluated.

**Table 1:** Description of power reflectance (PR) test in 105 newborns evaluated.

	Frequency	Passed the test		Failed the test	
		*n*	%	*n*	%
Right side	1000 Hz	91	86.7	14	13.3
	2000 Hz	77	73.3	28	26.7
	3000 Hz	89	84.8	16	15.2
	4000 Hz	95	90.5	10	8.5
Left side	1000 Hz	78	74.3	27	25.7
	2000 Hz	74	70.5	31	29.5
	3000 Hz	91	86.7	14	13.3
	4000 Hz	99	94.3	6	5.7

### Agreement between tests

Table 2 presents Kappa coefficients and respective confidence intervals amongst the three tests assessed together. There was a low agreement (*p* ≤ 0.05) among the results of tympanometry, TOAE, and PR at a frequency of 2000 Hz on the right side. With respect to the left side, a reasonable agreement (*p* ≤ 0.05) was seen among the results of tympanometry, TOAE, and PR (at 2000 and 3000 Hz). Table 3 shows the *κ* coefficient and the confidence interval between test results (taken two by two) for tympanometry and PR. For both sides, there was reasonable agreement between tympanometry and PR at 2000 and 3000 Hz.

**Table 2:** Kappa coefficient for agreement amongst TOAE, tympanometry, and PR tests in 105 newborns evaluated.

PR	Frequency	*n*	Kappa	95% CI	Classification	*p*-Value
Right side	1000 Hz	98	0.18	0.00–0.40	–	0.251
	2000 Hz	98	0.20	0.01–0.44	Low	0.047
	3000 Hz	98	0.17	0.00–0.45	–	0.126
	4000 Hz	98	0.13	0.00–0.45	–	0.201
Left side	1000 Hz	100	0.12	0.00–0.34	–	0.144
	2000 Hz	100	0.31	0.10–0.52	Reasonable	0.002
	3000 Hz	100	0.35	0.09–0.62	Reasonable	0.005
	4000 Hz	100	0.23	0.00–0.54	–	0.059

*n*, Cases with information on all three tests; 95% CI, 95% confidence interval; –, non-significant agreement.

**Table 3:** Agreement between tympanometry and PR exams in 105 newborns evaluated.

Tests	PR (1000 Hz)	PR (2000 Hz)	PR (3000 Hz)	PR (4000 Hz)
*Right side*				
Tympanometry	0.01	0.29	0.22	0.16
Confidence interval	(0–0.15)	(0.12–0.47)	(0.02–0.42)	(0–0.36)

*Left side*				
Tympanometry	0.04	0.32	0.37	0.17
Confidence interval	(0–0.20)	(0.15–0.49)	(0.18–0.56)	(0–0.35)

#### Tympanometry specificity

Table 4 presents the specific measures for tympanometry. Interpreting the examination of the curve for the right side, a specificity of 87.2% (78.4–92.9) was found. This indicates that 87.2% of those patients that passed TOAE had a normal result for the curve. Interpreting the examination of the curve for the left side, a specificity of 83% (73.5–89.7) was found. This indicates that 83% of those patients who have passed TOAE had a normal result of the curve.

**Table 4:** Tympanometry specificity in 105 newborns evaluated.

Side	Specificity
Right	87.2% (78.4–92.9)
Left	83.0% (73.5–89.7)

#### Power reflectance specificity

Table 5 presents the specific measures for PR test in the evaluated frequencies.

**Table 5:** PR test specificity in evaluated frequencies in 105 newborns evaluated.

Side	Frequency	Specificity
Right	1000 Hz	86.6% (77.8–92.4)
	2000 Hz	75.3% (65.3–83.2)
	3000 Hz	85.9% (77.1–91.8)
	4000 Hz	90.9% (83.0–95.5)
Left	1000 Hz	76.3% (66.4–84.1)
	2000 Hz	76.0% (66.0–83.9)
	3000 Hz	90.7% (82.7–95.4)
	4000 Hz	95.9% (89.3–98.7)

## Discussion

The importance of the NHSP is unquestionable, demonstrating that for every 1000 children examined, two to three (0.2–0.3%) will present inner ear disease, according to the Joint Committee on Infant Hearing^1^; and, according to Yang,^22^ 100 children (10%) will present conductive hearing loss caused by middle ear dysfunction.

Keefe^23^, ^24^ recommended a middle ear study in association with the newborn hearing screening using tympanometry and/or power reflectance, considering that hearing screening interpretation would be more appropriate.

### Descriptive analysis of TOAE, PR, and tympanometry results

In the overall assessment of TOAE in 105 children (210 ears), 92.4% passed in the right ear and 91.4% in the left ear; these results were similar to those described by Thompson.^25^

Tympanometry reveals the middle ear condition and is the most widely used test for this evaluation. In cases of conductive loss, altered tympanometry can be seen. In this study, using the tympanometry test, changes were observed in 14.9% of the neonates on the right side in and 20.6% on the left side; these are higher percentages compared to those published by Yang.^22^ It may be that these findings were found because the present study analyzed only the frequency of 1000 Hz which, according to the Joint Committee on Infant Hearing^1^ and the United Kingdom Newborn Hearing Screening Program,^26^ is the recommended tone for infants aged under 6 months. However, in the study by Yang,^22^ frequencies of 226 and 1000 Hz were analyzed. Nonetheless, it has been observed that tympanometry results obtained with a low-frequency probe tone (226 Hz) can be considered as normal, even in the presence middle ear changes.^27^, ^28^

PR measures the energy transferred passively to the middle ear and reveals the state of the external auditory canal and the TM. The results obtained from the evaluated frequencies showed that, at 1000 Hz, 13.3% of neonates failed on the right side and 25.7% failed on the left side; at 2000 Hz, 26.7% failed on the right side and 29.5% failed on the left side; at 3000 Hz, 15.2% failed on the right side and 13.3% failed on the left side; and finally at 4000 Hz, 8.5% failed on the right side and 5.7% failed on the left side. These results show that the frequencies of 2000 Hz and 3000 Hz were best suited for the determination of middle ear disease, agreeing with the findings of Roswski^29^ and Voss,^30^ who reported that when the value of PR is close to 1, at 2000 and 3000 Hz nearly all incident energy in these frequencies is reflected by TM, with little energy absorption by middle and inner ears. The maximum energy transmitted to the structures behind TM occurs when PR has its values between 0.3 and 0.4 at 1000 and 4000 Hz, which agrees with the results of this study.

Keefe^16^ examined PR measurement from 125 to 10,700 Hz in adults and in term neonates aged 1–24 months, and hypothesized that the frequencies of 2000–4000 Hz may be useful in clinical tests for middle ear evaluation, because these frequencies better absorb the sound energy that returns to the auditory canal. This finding was also reported by Piskorski et al.^31^; these authors stated that such frequencies can predict the presence of conductive hearing loss in children aged 2–11 years.

### Tympanometry and PR specificities

Using the results of newborns that passed TOAE as the gold standard in hearing assessment, tympanometry and PR specificities were calculated. The specificity of PR at all frequencies studied ranged from 75.3% to 95.9%. This shows that 5–25% of newborns have changes detected by PR not detected by TOAE. The specificity of tympanometry shows that 13–17% of newborns had changes with TOAE present.

These values are close to the 10% of conductive change prevalence in neonates described by Yang.^22^ One could question whether PR and tympanometry tests would be able to detect subclinical alterations in the middle ear that do not cause interference with TOAE capture.

As described by Hunter,^13^ WBR was significantly different in ears that failed *vs.* ears that passed the distortion-product otoacoustic emissions (DPOAE) testing, showing significant results to predict DPOAE status.

Evidence of a middle ear disorder as a major cause of ears that fail DPOAE tests is ascribed to the passive energy transfer that occurs in the middle ear, as measured by WBR, which when altered is related to middle ear disease, and not to cochlear function.

In addition, in children retested within days, in the face of WBR improvement, the percentage of success (*i.e.*, children who passed the test) also improved, showing a strong relationship between changes of status in DPOAE and WBR. No scientific study has yet been published with TOAE and RBL or PR as predictive value; however, considering that both DPOAE and TOAE are accepted as screening tests, this study was compared to the aforementioned study, and obtained similar results.

In this study, it was observed that PR values above pre-determined equipment reference levels in the Mimosa-type study^13^ suggest the presence of fluid in the middle ear.

These results are in agreement with those of Roswski^29^; that author reported that when the value of PR is close to 1 at frequencies of 2000 and 3000 Hz, almost all energy incident in these frequencies is reflected by the TM, resulting in little absorption of energy through middle and inner ear. The frequencies considered most suitable for middle ear disease detection were 2000 and 3000 Hz, because these frequencies show reasonable agreement with tympanometry at 1000 Hz (the most often used test for middle ear disease detection) and also for presenting the higher number of ears that failed PR. Similarly to the present results, Feeney and Sanford^32^ and other investigators found higher changes for PR value at 2000 Hz.

The maximum energy transmitted to the structures behind TM occured when PR values were between 0.3 and 0.4 at the frequencies of 1000 and 4000 Hz,^29^ which are similar to the results found in this study.

In the present study, it was noted that PR values above the reference levels suggest presence of fluid in the middle ear, and therefore a conductive alteration, a finding first described by Voss.^30^

## Conclusion

PR at 2000 and 3000 Hz correlated with the results of TOAE and tympanometry tests. Frequencies of 2000 and 3000 Hz were the most suitable for middle ear disease detection using PR. It was also noted that the finding of PR values above the reference levels suggest presence of fluid in the middle ear, and thus, a conductive alteration. The specificity of PR at all frequencies studied ranged from 75.3% to 95.9%, and of tympanometry at 1000 Hz, ranged from 83% to 87.2%.

This study pointed out the need for further study of the PR test, in order to validate PR as an appropriate tool for middle ear assessment.

## Conflicts of interest

The authors declare no conflicts of interest.
